# The computational pharmacology of oculomotion

**DOI:** 10.1007/s00213-019-05240-0

**Published:** 2019-04-13

**Authors:** Thomas Parr, Karl J Friston

**Affiliations:** Wellcome Centre for Human Neuroimaging, Institute of Neurology, University College London, 12 Queen Square, London, WC1N 3BG UK

**Keywords:** Active inference, Bayesian, Computational pharmacology, Neuromodulation, Oculomotion

## Abstract

Many physiological and pathological changes in brain function manifest in eye-movement control. As such, assessment of oculomotion is an invaluable part of a clinical examination and affords a non-invasive window on several key aspects of neuronal computation. While oculomotion is often used to detect deficits of the sort associated with vascular or neoplastic events; subtler (e.g. pharmacological) effects on neuronal processing also induce oculomotor changes. We have previously framed oculomotor control as part of active vision, namely, a process of inference comprising two distinct but related challenges. The first is inferring where to look, and the second is inferring how to implement the selected action. In this paper, we draw from recent theoretical work on the neuromodulatory control of active inference. This allows us to simulate the sort of changes we would expect in oculomotor behaviour, following pharmacological enhancement or suppression of key neuromodulators—in terms of deciding where to look and the ensuing trajectory of the eye movement itself. We focus upon the influence of cholinergic and GABAergic agents on the speed of saccades, and consider dopaminergic and noradrenergic effects on more complex, memory-guided, behaviour. In principle, a computational approach to understanding the relationship between pharmacology and oculomotor behaviour affords the opportunity to estimate the influence of a given pharmaceutical upon neuronal function, and to use this to optimise therapeutic interventions on an individual basis.

## Introduction

Oculomotor behaviour relies upon the coordination of a distributed network of regions throughout the brain (Parr and Friston 2017a; Robinson 1968). Assessment of oculomotion therefore offers a simple (non-invasive) way to measure brain function. While disruption of normal neurological (Anderson and MacAskill 2013) or psychiatric (Lipton et al. 1983) function can induce a range of characteristic eye-movement deficits; subtler modulations of neuronal function may also be detected in oculomotion. In this paper, we focus upon the neurochemical aspects of oculomotor control, and the sorts of oculomotor syndromes that may be induced by therapeutic agents (Naicker et al. 2017; Reilly et al. 2008). In doing so, we draw from recent theoretical work addressing the computational anatomy of oculomotion (Parr and Friston 2018a; Parr and Friston 2018c) and emerging themes in computational accounts of neuromodulation (Friston et al. 2014; Marshall et al. 2016; Parr et al. 2018; Parr and Friston 2017b; Sales et al. 2018; Schwartenbeck et al. 2015). These accounts are based upon the idea that the brain uses a generative model to infer the causes of its sensations, and that this model is equipped with beliefs^1^ about the precision (inverse variance) of the relationships between different kinds of latent (i.e. unobserved) variables generating sensory (i.e. observed) samples. The precision of a belief can be thought of as the confidence in that belief (as opposed to its content). As such, precisions are generally associated with neuromodulatory influences over synaptic gain (Feldman and Friston 2010; Marder and Thirumalai 2002; Nadim and Bucher 2014), as opposed to driving postsynaptic responses (i.e. modulating transmembrane conductance as opposed to depolarisation).

We have previously argued for an association (i) between acetylcholine and beliefs about how precisely hidden variables in the world give rise to sensory data, (ii) between noradrenaline and beliefs about how hidden variables in the present cause those in the future, and (iii) between dopamine and beliefs about how we will act upon the world (Friston et al. 2014; Parr and Friston 2017b). In what follows, we first provide an overview of oculomotion in terms of these three kinds of precision, their associated neurotransmitter systems, and active inference. We then introduce a simple delay period oculomotor task—of the sort used extensively in primate electrophysiological studies (Funahashi 2015). Through manipulating various precision terms, we will see that the resulting oculomotor syndromes reproduce those induced by pharmacological agents acting upon their associated neurochemical systems. The implication here is that if one can generate pathological eye movements from selective deficits in neuromodulatory systems in silico, it is possible to estimate these deficits using empirical observations, such as eye tracking [see Adams et al. (2016) for a proof of principle using slow pursuit eye movements].

### Active inference and oculomotor control

In this section, we briefly overview the neuroanatomical networks involved in ocular control, with a special focus on the synapses on which different neurotransmitters are thought to act. In describing this functional anatomy, one can associate these neurotransmitters with putative computational roles. The direct cortical control of eye movements involves predominantly dorsal brain areas, including the frontal eye fields (Künzle and Akert 1977), which communicate with the nearby dorsolateral prefrontal cortex (Buschman and Miller 2007). The former area is thought to represent the position of the eyes (Moore and Fallah 2001), while the latter is associated with the maintenance of beliefs about cued targets (Goldman-Rakic 1987). Like most of the cortex, these areas receive distributed projections from the locus coeruleus and the basal forebrain via the cingulum (Avery and Krichmar 2017; Doya 2008). As such, these cortical regions are modulated by noradrenaline and acetylcholine. Under active inference, noradrenaline is thought to represent the precision of transitions (i.e. confidence in probabilistic beliefs about the dynamics of the world—such as motion and occlusion). This encoding of precision is crucial in prefrontal cortical regions involved in the maintenance of a remembered stimulus, as the persistence of a belief over time rests upon a precise belief that the target does not change between viewing the stimulus and enacting the appropriate response (Parr and Friston 2017c).

Acetylcholine has been linked to the precision of beliefs about how latent or hidden states of the world—that cannot be directly observed (e.g. eye position)—give rise to sensory (visual or proprioceptive) data (Marshall et al. 2016; Moran et al. 2013; Vossel et al. 2014). Loss of acetylcholine, as observed in conditions such as Lewy body dementia, can lead to a failure of sensory data to constrain perceptual inference in the right sort of way. Complex visual hallucinations—namely, false positive perceptual inference (Collerton et al. 2005; Parr et al. 2018)—represent a dramatic example of this failure. In the context of motor control, imprecise predictions about desired movements may lead to a failure of descending predictions (i.e. motor commands) to elicit the predicted proprioceptive signals via motor reflexes.

**Fig. 1:**
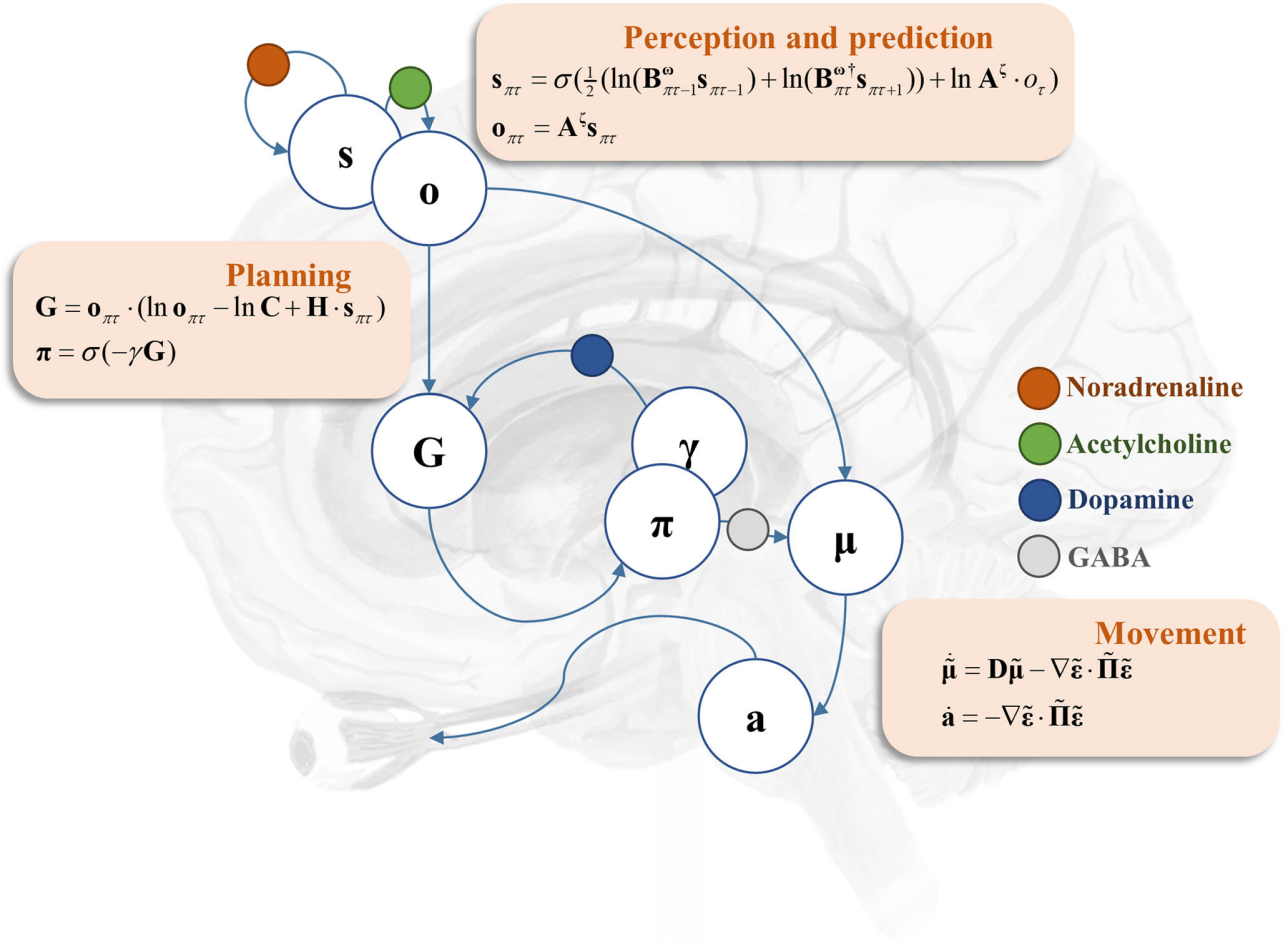
Computational neuropharmacology and oculomotion. This schematic illustrates a simplified (computational) anatomy of oculomotor control, highlighting some of the key synapses at which neuromodulatory transmitters act. The cortical components of this network include the frontal eye fields and the dorsolateral prefrontal cortex. We have associated these regions with beliefs about hidden states (**s**), and predictions about the (categorical) outcomes (**o**) that these states entail. The ‘perception and prediction’ panel specifies how these are computed from beliefs about the way in which states give rise to observations (**A**), and beliefs about how states at a given time evolve (**B**). These likelihood and prior transition terms are equipped with precisions—superscripts **ζ** and **ω**, respectively—that quantify the confidence (inverse variance) of associated conditional beliefs. The likelihood and prior precisions have been associated with cholinergic and catecholaminergic modulation respectively. These cortical regions project to both the basal ganglia (i.e. the striatum) and the superior colliculus. The direct pathway through the basal ganglia itself targets the superior colliculus, via the substantia nigra pars reticulata. Striatal neurons are modulated by dopaminergic projections from the substantia nigra pars compacta (**γ**), while the projections from the pars reticulata to the superior colliculus provide a GABAergic modulation of the cortico-collicular pathway (**Π**). The ‘planning’ panel shows how the basal ganglia may evaluate alternative saccades by computing the expected free energy (**G**) associated with each eye movement and subsequent sensory samples. Dopamine modulates confidence in beliefs about the best saccade to select (**π**), given this evaluation. The ‘movement’ panel provides the (Bayesian filtering) equations that may be used to implement the next saccade. These rely upon prior beliefs about where the eyes should be that are obtained from the predictions from the cortex (**o**) modulated by plans evaluated in the basal ganglia (**π**). Together, these are used to compute the average belief about where to look next, which is then equipped with a precision (**Π**). The error (**ε**) between current beliefs about the position of the eyes (**μ**) and the target is then used to drive brainstem reflexes that act (**a**) to minimise this error—implementing the motor command from the cerebrum. The form of these equations may look complicated; however, they can be derived in a fairly straightforward way from standard (variational message passing) schemes under ideal Bayesian observer assumptions. From our perspective, the key feature of these equations is that they suggest a modulatory role for the precisions described above. For a more detailed technical account of these equations, please see (Friston et al. 2017b), and for a conceptual overview of their relationship to anatomy, please see (Parr and Friston 2018b)

The cortical regions described above communicate with brainstem oculomotor regions via two main pathways. The first is a direct cortico-collicular projection (Künzle and Akert 1977). The second is via the basal ganglia (Hikosaka et al. 2000). The output nuclei of the basal ganglia include the substantia nigra pars reticulata, which monosynaptically inhibits the superior colliculus via GABAergic projections (Hikosaka and Wurtz 1983). The other part of the substantia nigra—the pars compacta—provides dopaminergic innervation to the striatum (Moss and Bolam 2008). In terms of active vision, the cortico-collicular pathways may be thought of as predicting the proprioceptive and visual consequences of alternative saccades that could be performed. The nigro-collicular pathway then weights each alternative, depending upon striatal evaluations of the ‘goodness’ (technically, expected free energy) of each possible saccade. This goodness is simply the capacity of that saccade to fulfil prior beliefs about the sensory outcomes of a visual sampling (e.g. to comply with experimental instructions or to resolve uncertainty by accumulating evidence during visual scene construction). This sets up a biased competition in the superior colliculus, resulting in the selection of a saccadic target (Veale et al. 2017; Zelinsky and Bisley 2015). The superior colliculus then propagates this signal to other oculomotor brainstem areas (Parr and Friston 2018a; Robinson 1968), resulting in a saccade towards this target. The GABAergic signal here is vital in setting up the competition between alternative saccades (Hall 1999), leading to a precise representation of the chosen saccadic target. Finally, in active inference formulations, the nigro-striatal pathway is responsible for maintaining precise beliefs about which saccadic policy to pursue. Please see Fig. 1 for a description of this computational anatomy in terms of Bayesian belief updating and neuronal message passing.

### Delayed oculomotor task

**Fig. 2:**
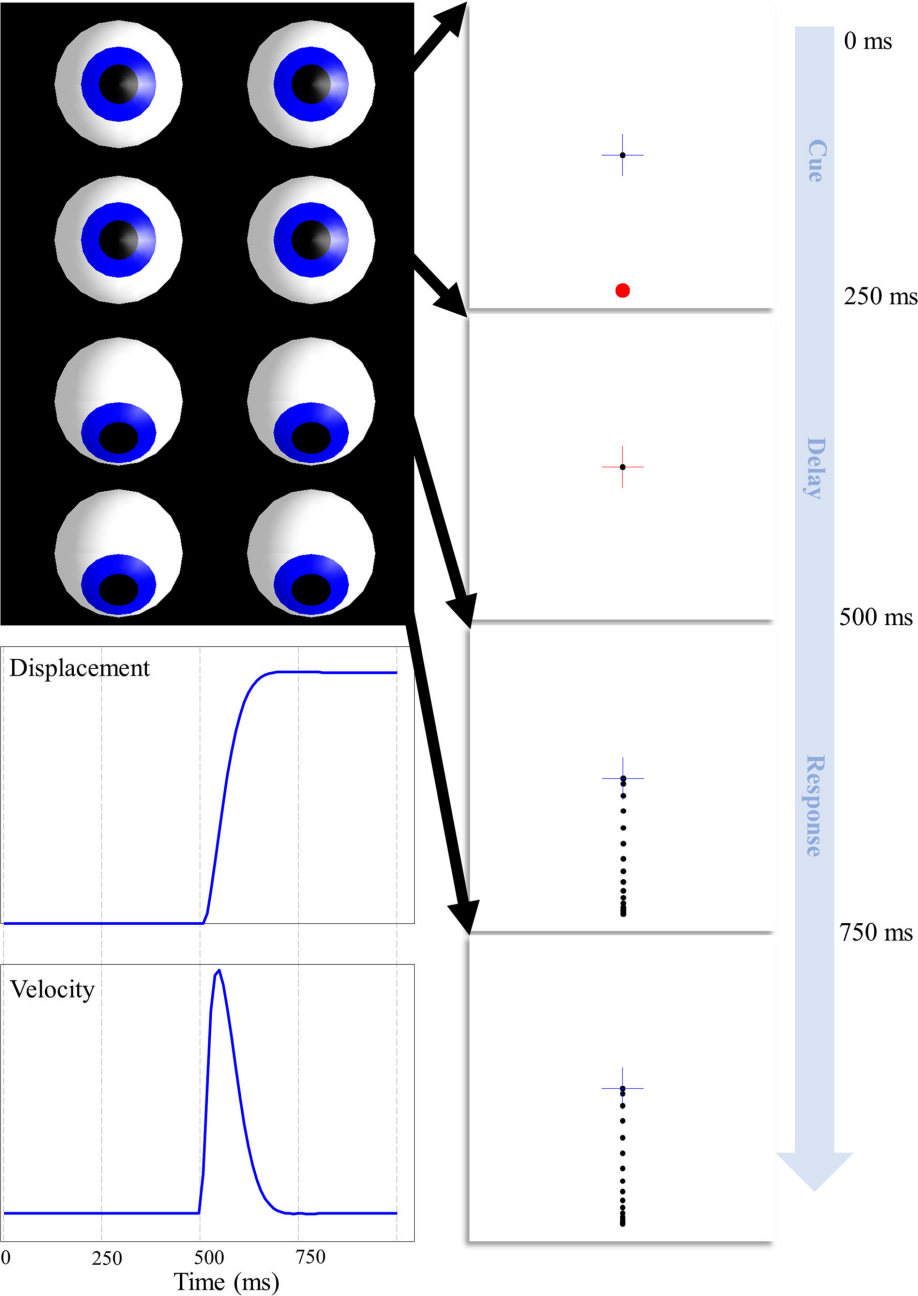
Delayed oculomotor task. This illustrates the sequence of a single trial of our simulated task. The task begins with fixation in the centre, while a peripheral target is presented. The target then disappears, but the cross changes to red, indicating that fixation must be maintained. When the cross changes back to blue, this indicates that a saccade should be made to the target location. The sequence shown represents correct task performance, where the saccade is withheld until the appropriate time, and is then directed to the correct location. The lower left panels show the displacement from the fixation cross and the velocity of the eyes as a function of time, while the upper left images show the position of the eyes at the end of each discrete time-step (i.e. the dotted vertical lines in the lower left plots). Note that the trial sequence takes place over four time-steps that each represents a 250 ms continuous trajectory. This was chosen for consistency with the frequency of saccadic sampling. While much longer delay periods are normally employed in practice, the model could be extended to deal with these simply by adding in additional delay periods (each of 250 ms). The dashed vertical lines in the plots on the lower left indicate the phases of the trial, as outlined here. These will be used in all subsequent figures for to aid comparison

A range of oculomotor changes have been observed following different therapeutic interventions. These include changes in the characteristics of a saccade (e.g. hypo or hypermetric), and in the decision processes leading to a saccade (e.g. inappropriate saccade targets). For the purposes of this paper, we adopt a single oculomotor task (Funahashi et al. 1989) that showcases a simple decision process but also allows us to inspect the trajectory of the saccade itself (Fig. 2). This involves presentation of a cue at the saccadic target location, followed by maintenance of fixation after the disappearance of the target. When cued, the task is to saccade to the remembered target. This oculomotor delay period task has been used extensively in primate research, notably in the study of working memory, so has well-described neurophysiological correlates.

To simulate oculomotor performance under this paradigm, we have to specifying a model of how sensory outcomes are generated by latent or hidden states of the world—and how those states can be changed by selecting particular actions or movements. This generative model then specifies belief updating in a synthetic brain under ideal Bayesian assumptions (see the equations in Fig. 1). The beliefs in question here are expectations about states of the world generating sensations—and the plausible actions that can change those states. Crucially, the synthetic subject believes she will select those actions that maximise the evidence for her model of the world. It transpires that this is the same as selectively sampling in sensory outcomes (e.g. directing saccades to particular parts of the visual field) that resolve uncertainty. This resolution of uncertainty comes in two flavours. First, the information gained by sampling new observations and, second, ensuring that these observations are consistent with the generative model (i.e. conform to prior preferences). In the following generative model, we have to deal with two sorts of states, namely, discrete and continuous states. Discrete states correspond to different locations, different stages of each trial etc., while continuous states refer to things like eye position and velocity.

The generative model we use to simulate this task uses a Markov decision process (MDP) model of discrete states (Friston et al. 2015) that generates a set of hypothetical saccadic targets. Each of these hypotheses represents the equilibrium (attracting) point (Feldman and Levin 2009) in a continuous state-space model (described in detail in (Parr and Friston 2018a)) of the eyes themselves. The MDP part of the model comprises three types of hidden (unobservable) state—that jointly generate discrete predictions for the continuous part of model dealing with continuous oculomotor trajectories (Friston et al. 2017b). The discrete states generating predictions include the current fixation location, the target location, and the current stage of the trial. The last of these states includes the target presentation, delay period, and saccade-to-target stages. Fixation location is a controllable state, meaning that any of the five (fixation cross, up, down, left, right) locations may change to any other location depending upon the saccade selected. The target location is static over time, ensuring it is the same at the end of the trial as it was at the beginning. It is this enduring context that gives rise to the delay period activity—thought to be supported by recurrent glutamatergic connections in Layer III of the cortex (Kritzer and Goldman-Rakic 1995)—characteristic of the prefrontal cortex. The third hidden state models transition to the next stage of the trial, at each time-step.

During the fixation stage of the trial, the visual outcome indicates the target location. During the delay period, the cross turns red and no cues are shown. An outcome ‘incorrect’ occurs if a saccade is performed during this step. A priori, this outcome is not preferred and is therefore avoided. At the final stage of the trial, the cross changes from back to blue, and a saccade is permitted. ‘Correct’ outcomes ensue if the fixation location hidden state at this time matches the target location, and ‘incorrect’ pursues otherwise. Prior preferences dictate that ‘correct’ outcomes mark a particular saccade as more likely (in terms of maximising the model evidence or minimising free energy expected following a saccade). At all stages, the proprioceptive outcome is generated through an identity mapping from the fixation location (i.e. the subject has precise sensory evidence about where she is looking). We do not explicitly model free free-viewing during inter-trial intervals and assume that feedback is given immediately following the response. Equipped with this model, we can now examine the effects of changing the precisions (i.e. simulated neuromodulators) on task performance.

### Gamma-aminobutyric acid

**Fig. 3:**
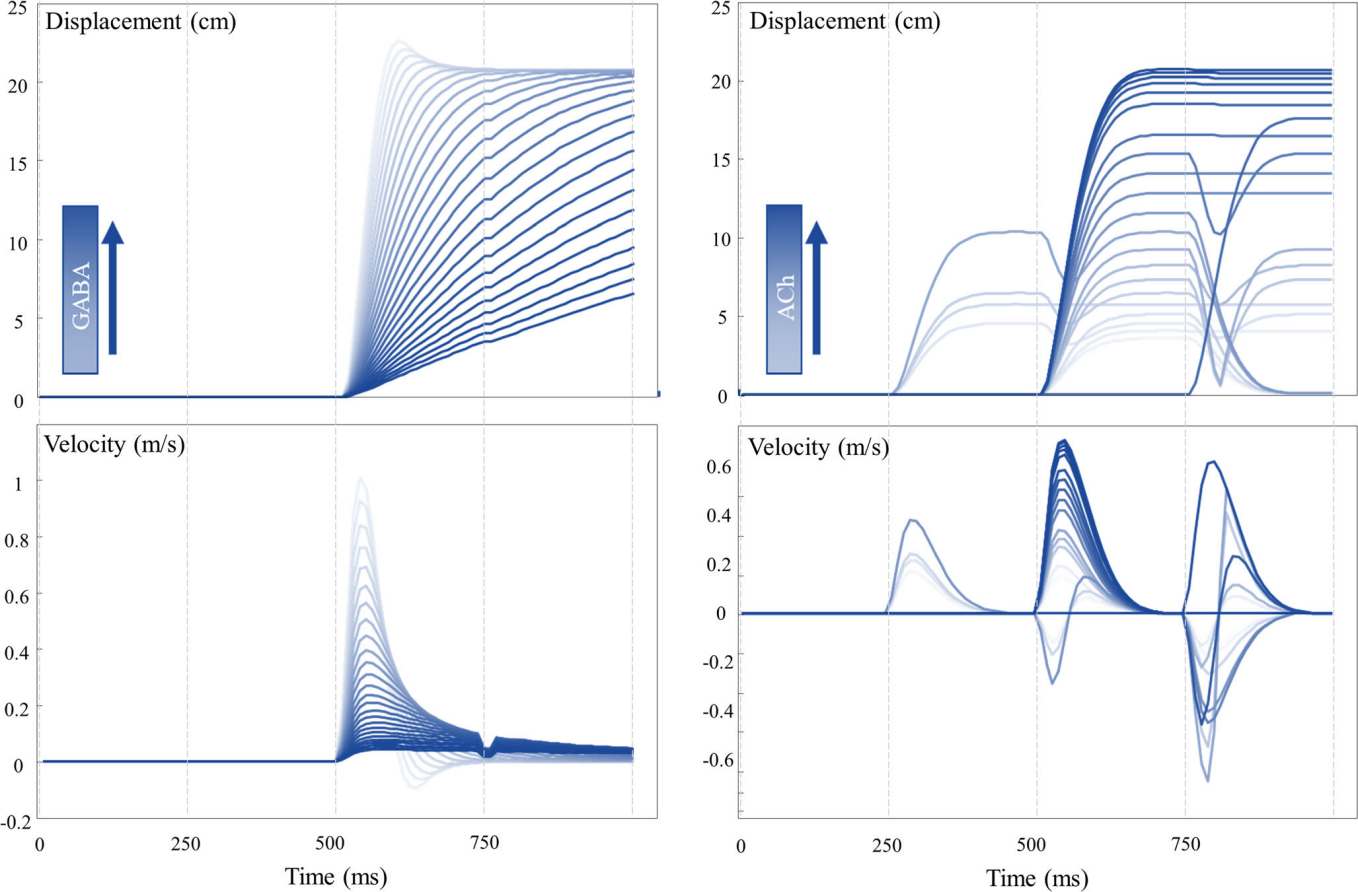
Saccade characteristics. The plots on the left show a set of trials with varying levels of GABA (**Π** from Fig. 1). The upper plot shows the displacement over time through the trial, while the lower plot shows the associated velocity. Note that very low levels of GABA result in an overshoot, that is subsequently corrected, and a higher velocity. In contrast, high levels of GABA lead to slow, hypo-metric saccades. These become broken when the velocity is sufficiently slow that the saccade takes more than one discrete time-step (vertical dashed lines) to complete. The plots on the right show the same characterisation of saccades with varying levels of cholinergic modulation (**ζ** from Fig. 1). These show a similar, but inverted, phenomenology; with increasing levels of acetylcholine leading to faster saccades. Unlike with the GABAergic changes, there is no hypermetric overshoot. The saccades instead converge to the optimal distance. At low levels of acetylcholine, saccades start to occur too early or late, and in some cases, more than one saccade occurs during a given trial. It is useful to try to infer where the normal physiological range of these parameters may lie—to understand the difference between overdoses or depletions. While this is really an empirical question, best answered by fitting these models to data, we can try to address this issue heuristically. Given that healthy eye movements tend not to overshoot, and that they reach their target displacement quickly, this suggests normal physiological ranges are at the lower end of the GABA scale, and that most of the traces shown above represent excesses above this (with the exception of those that overshoot, which may be depleted). Similarly, the relatively low frequency of inappropriate saccades in healthy people suggests that physiological ranges of acetylcholine are at the higher end of the scale shown here. The improvement elicited by some cholinergic drugs (see main text) suggests that the normal range is not quite at the higher limit shown here

Benzodiazepines are a class of pharmacological agents that act through modulation of GABAergic activity (Griffin et al. 2013). Specifically, they bind to the GABA_A_ receptor and facilitate action of the endogenous neurotransmitter. They are commonly used in clinical practice to treat a range of conditions including, but not limited to, anxiety disorders, insomnia, and (in an acute setting) epilepsy. Oculomotor changes during use of these agents are sufficiently robust that they have been proposed as biomarkers for the pharmacological effects (de Visser et al. 2003). These effects include a clear (inverse) dose-response relationship (Bittencourt et al. 1981) with saccadic peak velocity. Although the actions of systemic benzodiazepine administration are difficult to localise, this has also been demonstrated using anatomically precise injections of muscimol (a GABA agonist) directly into the superior colliculus (Hikosaka and Wurtz 1985). This induced a similar attenuation of saccade peak velocity. This is consistent with Fig. 1, which associates the oculomotor effects of gamma-aminobutyric acid (GABA) with inhibition of the superior colliculus, and with Fig. 3, which shows the effect of increasing the precision of beliefs about the anticipated eye position (empirical prior) on the displacement and velocity of a saccade over time. Notably, the peak velocity decreases with increasing precision, consistent with the effect of increasing the dose of a benzodiazepine. Intuitively, the greater the prior precision is over the dynamics represented in the brainstem, the harder it is to update these beliefs such that the eyes can move to a new location.

### Acetylcholine

Cholinergic or anticholinergic effects are common to many drug classes (Campbell et al. 2009; Ness et al. 2006) and also represent an important mode of action of several toxins (e.g. organophosphate pesticides (Minton and Murray 1988)). As with the benzodiazepines, cholinergic effects have been associated with the velocity of a saccadic eye movement (Naicker et al. 2017). This is interesting from the perspective of the scheme in Fig. 1, as cholinergic modulation is hypothesised to occur at the level of inference about categorical variables (which saccade to perform and which location is the target). It is not immediately obvious how such inferences could influence continuous variables such as velocity. In addition to its cortical site of action, acetylcholine is vital in the normal function of the striatum and has actions on brainstem nuclei directly (Dautan et al. 2014; Kobayashi and Isa 2002; Maurice et al. 2015). The cholinergic (precision) manipulations illustrated in Fig. 3 suggest that these categorical inferences can influence velocity in a consistent way. As cholinergic transmission increases so does the peak saccade velocity. This is an example of a functional diaschisis (Carrera and Tononi 2014; Fornito et al. 2015; Price et al. 2001), in which altering one part of a network has implications for all other parts. The effect here is due to the fact that the precision of the state-outcome mapping determines the precision of the predictive distribution over alternative saccadic locations. If this distribution becomes less precise, the expected (average) anticipated location in continuous coordinates becomes a mixture of all of the possible locations, weighted by their relative probability. When the precision is low, this means saccades towards more central locations become more probable and that, as precision increases, the anticipated location will move further towards a specific target.

Hyoscine (a.k.a. scopolamine), an antimuscarinic (anticholinergic) drug (Corallo et al. 2009) used to treat motion sickness, slows the velocity of saccades (Oliva et al. 1993), consistent with Fig. 3. Interestingly, it additionally causes saccades to become hypo-metric and impairs the stability of fixation. These effects are shown clearly in Fig. 3, with lower levels of cholinergic signalling leading to shorter saccades, and an increase in the number of inappropriate saccades. The latter are due to the fact that precision sharpens or flattens the distribution of plausible saccadic targets. As this distribution becomes flatter, saccades previously deemed inappropriate acquire plausibility. Agonists of the cholinergic system, notably nicotine, improve the performance of saccade (specifically ‘anti-saccade’) tasks if performance is suboptimal (e.g. on first exposure to a task) (Rycroft et al. 2006). These effects tend to saturate fairly quickly, implying that nicotine adds no additional benefit (or deficit) when task performance has already been optimised—and this may be why some studies report no improvement or changes in velocity with nicotine administration (Sherr et al. 2002). This is consistent with the saturation of responses we see in our simulations, with increasing levels of precision asymptotically approaching optimal saccadic trajectories. It is encouraging that these studies show similar results to the computationally focal manipulations performed in our simulations, despite the fact that these drugs do not act in an anatomically specific way.

### Dopamine

**Fig. 4:**
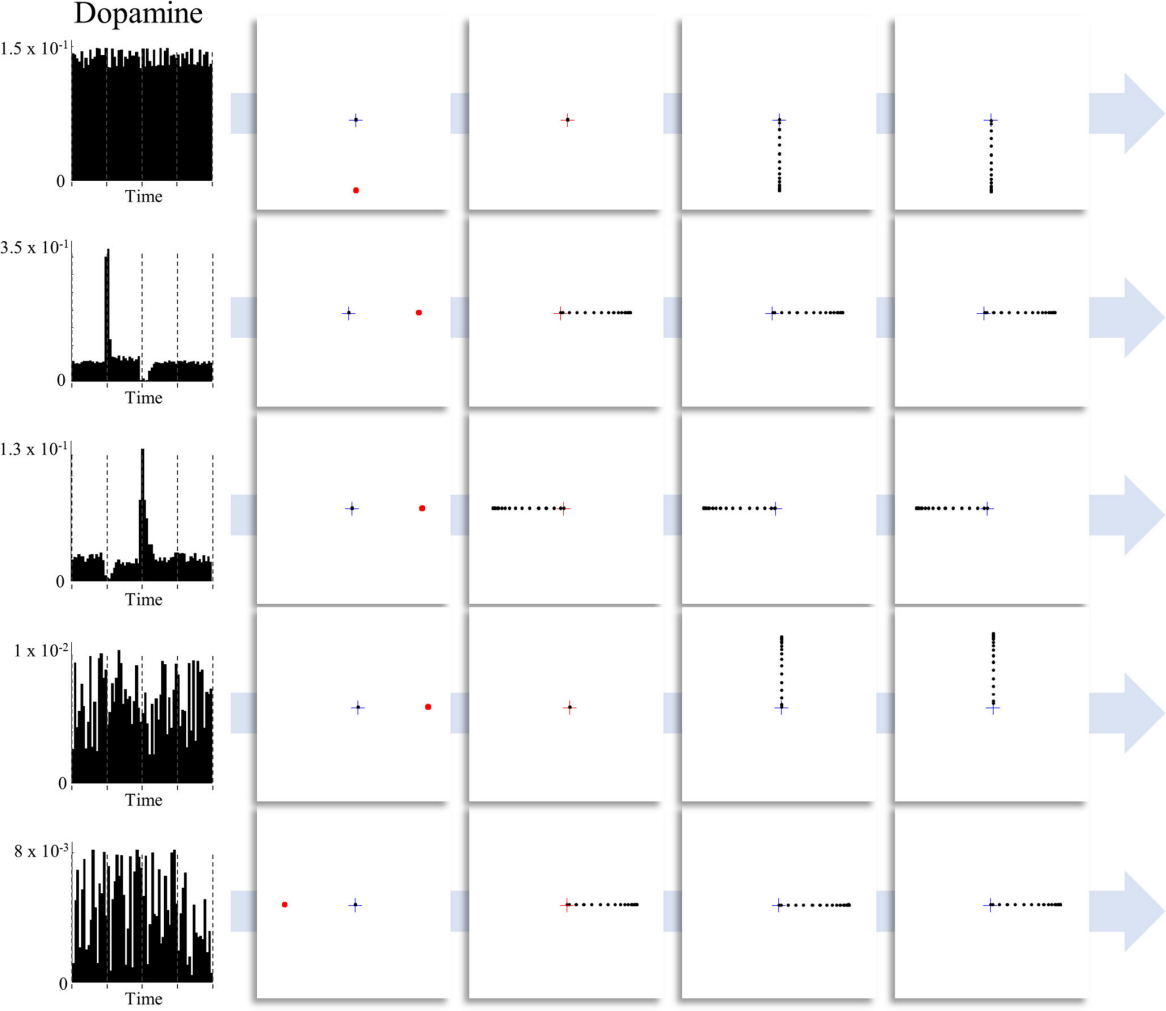
Dopaminergic modulation of saccadic choices. This figure illustrates the effect of dopaminergic modulation of decision making during the delay period task. Each row shows a different level of prior precision over policies (highest for the first row and lowest for the last). Simulated dopaminergic firing rates are shown on the left (note the differences in axis ranges). When the precision is very low, the selected saccadic target is random, as all possible saccadic policies become (nearly) equally probable. As the prior precision is increased, the first notable change occurs in the dopamine plots (third row). Here, there is a decrease in dopamine firing during the first saccade, as uncertainty about the policy pursued increases. This is because this saccade is inconsistent with the policy consistent with reaching the target. Having committed to the incorrect policy, there is a dopamine spike coinciding with a confident inference that this policy is being pursued. As dopamine levels increase further, this spike moves earlier, as the saccade performed (although still premature) is still consistent with reaching the target. When sufficiently high, dopamine levels show little change throughout the trial, with the correct policy inferred quickly and confidently from the first time-step. The key message to take away from this figure is that, in the absence of dopamine, oculomotor decisions become increasingly random. This is because the distribution over action sequences becomes less precise. As the precision tends towards zero, all plans of action become equally probable

Pharmacological manipulation of the dopamine system can be highly effective in treating both neurological and psychiatric disorders. Parkinson’s disease, in which the substantia nigra pars compacta degenerates, responds to L-Dopa (a dopamine precursor) (Smith et al. 2012), in addition to dopamine agonists (Jenner 1995). In contrast, suppression of dopaminergic activity is a key part of the pharmacological strategy adopted in antipsychotic medications (Kapur et al. 2000). In oculomotor tasks, impairments in dopamine signalling cause deficits in saccades, most pronounced in memory-guided saccades (Kato et al. 1995; Kori et al. 1995). Similar deficits have been described in Parkinson’s disease (Chan et al. 2005), in which there is a degeneration of dopaminergic nuclei. This is highly consistent with the active inference account of dopamine as representing the precision of beliefs about temporally deep policies, or plans about how to act (Friston et al. 2017a). While a visually guided saccade requires a planning depth of one-step-ahead, a memory-guided task required the inference of the appropriate plan over multiple time-steps (from presentation of the cue to the execution of the action). We have previously demonstrated the influence of dopamine on simulated saccades in a memory-guided paradigm (Parr and Friston 2017c), and here replicate this influence in the oculomotor delay period task described above. Figure 4 illustrates the effect of changing the prior precision on simulated dopamine firing (updates in the precision over time) and its behavioural consequences. Memory-guided saccades are disrupted once dopamine levels drop. Not only are saccades performed to incorrect locations, they also occur at inappropriate times, consistent with the impairment in sequential planning induced here.

These simulations provide further face validity to the idea that dopamine is involved in signalling the precision of beliefs about deep policies. Previous theoretical accounts have reproduced aspects of the phenomenology of dopamine signalling based upon this (Friston et al. 2013) and have inspired empirical studies, including the use of simulated precision updates as regressors in functional imaging studies (implicating the dopaminergic midbrain) (Schwartenbeck et al. 2015), and modelling of behavioural responses under pharmacological manipulations (Marshall et al. 2016). A simple experiment that could be performed to further test these ideas would be to fit the model described here to the (saccadic) decisions made in this task (Mirza et al. 2018) and to see whether the prior precision over policies estimated from real participants correlates with their spontaneous blink rate—a peripheral manifestation of central dopamine function (Karson 1983).

### Noradrenaline

**Fig. 5:**
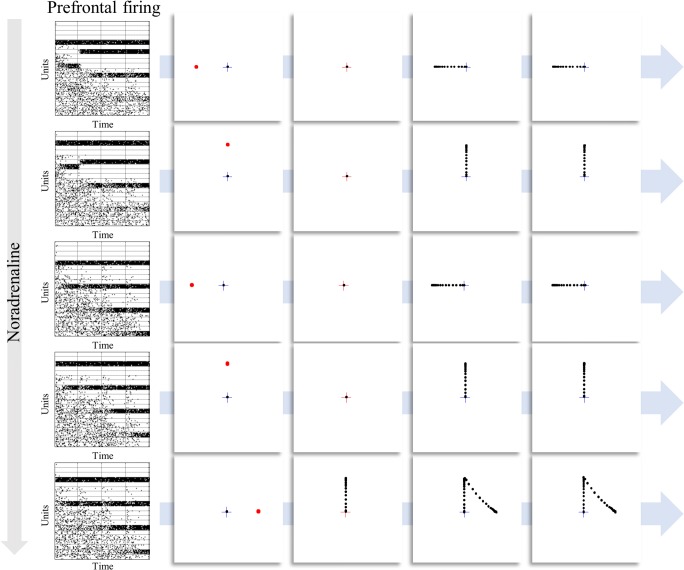
Noradrenergic modulation of prefrontal firing. Each row illustrates a single trial of the delay period oculomotor task, but with different levels of noradrenaline. This has little effect on the performance of the task. Even in the lowest row, where a premature saccade takes place, this mistake is corrected at the next time-step. Note that this error is a consequence of the random sampling of actions from beliefs about policies and does not occur on the majority of trials. It has been retained here to illustrate the change in strategy that leads to the successful completion of the task. While there are no clear behavioural consequences of this manipulation, the physiological implications are much more striking. These are shown as raster plots of prefrontal cortical neurons representing the remembered target location. Each row represents the firing of a population of neurons representing the probability of one of the target locations at specific times throughout the trial. The lower rows within these plots indicate later times. This means that at the first time-step (first column), the last row represents beliefs about the future. By the final time-step (last column), the last row represents beliefs about the present. As the concentration of noradrenaline increases, more structure becomes apparent in the lower parts of the plots, indicating a more successful propagation of the inferences drawn from observing the initial cue to the later points in the trial. Note that the increase in persistent activity is accompanied by a decrease in the firing of other neurons, representing the probability of alternative states

The evidence for a modulation of oculomotor responses by noradrenaline is less clear (Reilly et al. 2008). Although some saccadic tasks are reported to vary with noradrenergic modulation (e.g. using methylphenidate (Klein et al. 2002; O’Driscoll et al. 2005)), including upon the timing of saccades (Suzuki and Tanaka 2017), there is little evidence for a systematic influence over behaviour in the delayed oculomotor task. Despite this, there is evidence for neurophysiological changes in circuits implicated in task performance when prefrontal α2-adrenoreceptors are modulated (Arnsten 2011; Arnsten and Li 2005; Sawaguchi et al. 1990). Specifically, administration of clonidine, an α2-agonist, facilitates the delay period activity associated with maintenance of working memory (Li et al. 1999; Suzuki and Tanaka 2017). This is highly consistent with the simulations shown in Fig. 5, where increasing noradrenergic signalling improves the propagation of information about the past to the future (columns of the raster plots).

Intuitively, an inability to project precise beliefs into the future, or from the past to the present, should undermine the performance of this task. Under active inference, a simple explanation for the preservation of performance even in the absence of precise beliefs about transitions rests upon the use of deep (sequential) policies. If we are able to infer, based upon early observations, the course of action we will pursue, performance becomes robust to the degradation of memories about those observations. In other words, if I know I have to perform a saccade to the left location, whether I believe that this is the target location or not has no influence over my task performance. This illustrates the dissociation between beliefs about states of the world and beliefs about ‘how I will act’. This explanation, and the redundancy it implies, lends itself to an empirical hypothesis. Under a noradrenergic blockade, the sensitivity of task performance to dopamine depletion should increase. Similarly, increasing noradrenaline or dopamine signalling should be able to rescue impaired performance due to depletion of the other transmitter. This sort of hypothesis, concerning an interaction between precision terms, highlights an important future research direction. While, for simplicity, we have highlighted the behavioural or physiological responses that build an intuition about the role of precision parameters, these quantitative behaviours also enable us to plot the responses shown above alongside any of the manipulations employed here. This affords an opportunity to investigate the interaction between neuromodulators and the influence of, for example, noradrenergic modulations on dopamine firing.

Interestingly, selective noradrenaline uptake inhibitors have been shown to be efficacious in treating anxiety disorders (Montoya et al. 2016). These drugs increase signalling at α2-receptors (Grandoso et al. 2004), implying that anxiety may be partly mitigated through an induction of the belief that environmental dynamics are more precise. Under the view that stress is a manifestation of uncertainty (Peters et al. 2017), this conclusion makes a great deal of sense. The reduction in uncertainty about what will happen next, by setting up a belief that the world is actually quite predictable, may be an important part of the computational mechanism of action of these pharmacological agents.

## Discussion

In the above, we have demonstrated the face validity of the use of active inference to simulate the effects of pharmacological therapies on oculomotor behaviour. Previous accounts of these behaviours have proposed their utility as biomarkers for the action of therapeutic (or toxic) agents in individual patients (de Visser et al. 2001; de Visser et al. 2003; Reilly et al. 2008). Complementing this approach, we offer a mechanistic (computational) account that bridges the gap between chemical and behavioural changes. The advantage of casting this in computational terms is that the model used here can be fit to empirical (eye-tracking) data to estimate the changes in precision brought about by specific drugs (Adams et al. 2016; Mirza et al. 2018; Schwartenbeck and Friston 2016). This offers a mechanistically informed method for non-invasive evaluation of synaptic function in individual patients. In doing so, it may be possible to titrate drug doses to achieve an optimal change in central nervous system function or to avoid adverse psychopharmacological effects.

Part of the strength of this method is the appeal to behaviours that depend upon inferences in two different, but connected domains (Parr and Friston 2018c). These are categorical decisions between alternative saccadic targets that depend upon working memory and delay period activity, and the continuous implementation of these decisions through oculomotion. This means that it is possible to draw inferences, based upon behaviour, about the function of anatomically disparate brain regions using a single model. An important caveat here is that the model we have used is overly simple from a pharmacological perspective. Notably, we have neglected the fact that neuromodulatory compounds act at many different anatomical sites and have different effects on different receptor subtypes. These omissions undoubtedly have important computational consequences. This may be why, although we have captured some aspects of the oculomotor changes induced by different drugs, there are others that are not reproduced. For example, the changes in speed of saccades associated with dopaminergic changes (Lynch et al. 1997) were not seen here. Despite these limitations, the correspondence between drug effects and the behaviours resulting from changes to precision parameters adds further weight to computational accounts of neuromodulatory systems and offers a tool to evaluate these theoretical accounts empirically.

## Conclusion

This paper offers a computational perspective on the influence of commonly used drugs on behaviour. We considered oculomotor behaviour as a specific example that is known to vary with drug administration, and that is easy to measure with non-invasive techniques. The simulations presented above illustrate that some of the key features of oculomotor responses to pharmacological interventions can be replicated in silico through an appeal to active inference. This rests upon the idea that planning is inference about how to act, and that these inferences entail predictions about the sensory consequences of action. Each stage of this process is sensitive to the precision associated with the relationship between different kinds of variable, and these precisions are thought to manifest biologically as synaptic gain—subject to neuromodulatory chemicals. Ultimately, we hope that this approach will be useful in a clinical setting, enabling quantitative characterisations of pharmacologically induced synaptic modulations using non-invasive measures.

### Software note

Although the generative model changes from application to application, the belief updates described in this article are generic and can be implemented using standard routines (here spm_MDP_VB_X.m). These routines are available as Matlab code in the SPM academic software: http://www.fil.ion.ucl.ac.uk/spm/. Simulations of the sort reported above can be reproduced (and customized) via a graphical user interface by typing in >>DEM and selecting ‘Decisions to movements’ and associated demos.

## Footnotes

1 In this article, beliefs are used in a non-propositional sense to denote a posterior probability distribution. In other words, beliefs are used technically in the sense of Bayesian belief updating and belief propagation.
